# An International Dental Congress

**Published:** 1890-02

**Authors:** 


### EDITORIAL.

### An International Dental Congress.

It would seem from some intimations that have found their way to the surface, that there is a desire, on the part of some, at least, to have an International Dental Congress during the year 1892, the year it is now proposed to hold the grandest International Exposition that the world has ever seen. But as neither the time, place, nor absolute certainty of the occurrence of the latter has been settled, it might be well to wait a little before taking any active measures; the fact of holding some sort of a dental convocation during the year of 1892, if the International Exposition should be held, has doubtless been in the minds of many, but nothing as yet can be definitely settled upon in reference to the matter.

The New Jersey State Dental Society at its late meeting, took up the matter (the first to do so, so far as we know), and passed a resolution, and extended an invitation to other dental Societies throughout the United States. Now, this was a very easy thing to do, but was it the best thing? Certainly no State Dental Society can assume to be the representative body of the profession of the United States; there are more than thirty other

Societies in the country that in this respect stand on a level with the New Jersey State Society, at least it is presumed so; and it is doubtful whether a dozen of them would or could respond to such a call as this, and with anything like such a representation of the profession it would be unwise to take any definite action. There are two organizations in the country that far more fully represent the profession, than it is possible for any State Society to do, viz: The American and the Southern, and there are two others, that in one sense are above any State Society, viz: The Association of College Faculties and that of the Boards of Examiners. If these bodies should, after conference, think it best to call a meeting of the State and local societies to consider this question, it certainly ought to have more influence and receive more attention than if it came from a State or local society.

And furthermore, there are but two or three State societies that meet between this and the 8th of April next.

In view of all these things would it not be well to hold on a little, until we can hear more and know more, and till the profession generally can have an opportunity to consider more fully what ought to be done. It is sometimes a good thing to take time by the forelock, but then it is better to know that one can hold on—don't take hold of two or three hairs and have them pull out.

